# Hospitals

**Published:** 1876-12

**Authors:** 


### Hospitals.

### COOK COUNTY HOSPITAL, MEDICAL DEPARTMENT.

Service of Dr. Lyman.

1. *Pleurisy with Effusion; Aspiration.*

May 12, 1876. A tall young Norwegian, aged twenty-two, took cold two weeks ago, and suffered from sharp pains in the left side. A fluttering movement of the epigastrium indicates a displacement of the heart downward and inward. There is a slight increase of elevation on the right side of the chest. He has a cough, caused by bronchitic inflammation. The left side is dull below a line level with the nipple, but when he lies down resonance returns in front, and the posterior surface becomes dull throughout. The left side of the chest is just half full of fluid. It is a question whether diuretics, cathartics or sudorifics make much impression on collections of this kind. We get more effect in inflammatory diseases from alteratives and tonics. It will be best to remove the fluid without delay. Three days is long enough to wait, for as the lung is driven up it might be retained in its forced position long enough to become fixed. Deformity might then result from the collapse when the fluid should have disappeared. A hollow needle was introduced about two inches beneath the angle of the scapula, between the eighth and ninth ribs, and pushed in two and a half inches. The flow of serum began, then stopped, and did not start again until the needle was turned and the direction changed a little. Dr. L. remarked that the lung, as it unfolds on the retreating liquid, might touch the needle and excite cough. The entering of air into the expanding portions may also excite cough. If the needle should penetrate some vital organ, as the liver, the spleen, or the lung itself, no harm would follow these punctured wounds. If the effusion should have become purulent, it will be necessary to make a free incision, allow a free escape, and use an injecting fluid. It will be easy to tell whether the case is one of serous effusion or not, for on the one hand we shall have a clear history of an acute attack and the progressive distension of the sac; but if it is a case of empyema, the constitution will have been broken down. The inevitable hectic in these cases will indicate their character. Two quarts of a straw-colored liquid were obtained by the operation, showing how great the loss of strength must be from the deprivation to the blood of albumen, with which the contents of the jar were charged. The needle was withdrawn without pain or hæmorrhage. A bit of adhesive plaster was stuck on the wounded surface, and opium was ordered to allay irritation. It was remarked that the needle should be carefully disinfected by drawing carbolic acid through it. The heart now appeared near its normal position.

2. *Hemiplegia and Coma through Embolism.*

May 16. A middle aged man, admitted for acute rheumatism of the right shoulder. Yesterday, while he was perfectly conscious, the left arm began to have a rigidity. He could answer questions, though he was unable to put out the tongue. There was loss of motion first, then partial loss of consciousness, lapsing into complete paralysis and coma. We found a loud murmur of the heart had made its advent, and we suspect that the pain complained of a few days ago was due to the rheumatic invasion. The murmur indicated a fibrinous exudation on the valves. A portion of this we suppose has been washed off and lodged in the right middle cerebral artery. The brain was not deluged, as in apoplexy, but the supply of blood was cut off. The effect was gradual; the surface was the first to feel it. Such cases are not immediately fatal. The particle of embedded matter may be small, and we may have only softening, followed by fatty degeneration. It may afterward become capsulated, as in slight apoplexy. The patient died on the 17th. Large vegetations were found on the mitral valve, and an embolus was discovered on the right side of the brain, at the bifurcation of the middle meningeal artery.

3. *Tubercular Peritonitis.*

A young Swedish machinist, injured by lifting too much, as he says. Last fall he coughed and spat blood. Since then these symptoms have ceased entirely. He is now greatly emaciated; pulse rapid; temperature 99½°; abdomen distended and tender. There is abundant resonance over the left lung and the lower half of the right. There is reason to believe that the first step in consumption consists of inflammation ot the bronchioles in scrofulous persons. The lymphatic vessels open in the terminal branches of the bronchial tubes. With new products come new processes. The lymphatic glands grow by the deposit of a new material. Their enlargement causes pressure, sets up secondary bronchitis, awakes cough. Finally the tubercular mass which has arisen, becomes crowded together, and its elements begin to die. The surrounding tissues becoming infiltrated, break down in turn, and are cast off. This is different from pneumonia, and the explanation is different from the ideas of text books for the last half century. As tubercular deposits in the lung involve not the primary tubes, not the air cells, but the lymphatics, so we may have considerable progress in the formation of tubercle without cough. It is evident that in the case before us the continuance of the tubercular symptoms only from last fall is not sufficient to account for the patient's condition. The progress of his disease has been latent in the lungs. Some of the symptoms of consumption are secondary. Let us see as to his digestive system. The tongue is clean, but he has been troubled with vomiting. Pressure over the hypogastric region causes pain. From a spot above Poupart's ligament, clear across the abdomen, there is a hard nodular mass to be felt. The probability is that it consists of enlarged lymphatic glands. Tubercular matter has been deposited, increasing their volume and producing peritoneal inflammation. A case like this will never live long enough to have serious inroads made upon the lungs, unless by deposit of miliary matter, which causes rapid infiltration. A child may have a scrofulous inflammation of the ear; the glands behind the ear become tuberculous, and tubercular meningitis may be induced. There is therefore a striking resemblance to what takes place after inoculation of syphilis. It is important to distinguish between these different relations. Pneumonia is the least dangerous; tuberculosis is next so; amyloid degeneration is the most serious of all, destroying any and every organ in the body — three processes nearly related, yet their results far removed.

4. *Gonorrhœal Rheumatism.*

May 23. A slender young man says he had inflammatory rheumatism last summer, and was confined six weeks with a painful swelling of his ankles and knees. Ten weeks ago he contracted gonorrhœa. He has had the characteristic discharge ever since. Two weeks ago he began to suffer pain in the head, and afterward in the right hip and knee. On admission his circulation was 96; temperature 102°. He was ordered ten grains of salicylic acid every hour for two days. No relief of pain. Hereafter he will have ten grains of the iodide of potassium, in a fluid drachm of the tincture of guaiac, four times daily. We think salicylic acid has been given long enough. Generally two or three drachms are sufficient. So much has been given, but he is not relieved.

Gonorrhœal rheumatism is a result of a species of pyæmia. The fluid of the urethra sets up a poisoning of a peculiar character. With lying in women a low grade of uterine inflammation may cause mammary abscess. Articular inflammation even may occur in such cases. In the same way here. If it were simply rheumatic we should get relief by the use of corresponding remedies. The joints are not materially swollen to sight or to feeling, but he cries out pitifully when the leg is raised. We cannot in the description very well distinguish between this and a surgical disease, or between this and rheumatism. The most we can say is that it is an exquisitely painful affection. The duration may be two or three months. He must have opium, besides the iodide of potassium. We meet patients suffering from gonorrhœal neuralgia, affecting every part of the body. They have "rheumatism all over." The joints become so stiff they cannot be moved. Anchylosis may result. A man is reported as having acquired in this way anchylosis of every joint in his body. We have seen several individuals who, strange as it may seem, have more than once had such a disease from such a cause.

5. *Chronic Intermittent Fever.*

A well developed man had his first attack of ague in Indiana, last October. He had a relapse in March. Calomel and quinine broke up the fever then. Yesterday there was another relapse. The chill was followed by a pulse of 120 and a temperature of 104°. In typhoid fever the temperature is not so high at first. There is an impoverished condition of blood, a deposit of the *débris* of the blood in the liver, spleen, kidneys and brain. The functions of the nervous system are correspondingly disturbed. Alterations of the tissues of the body in consequence of hardship or exposure brings on a renewal of the attack. When the daily mean of temperature rises above 72°, the causes become prevalent, whatever they may be. Some say germs, others vicissitudes of temperature. The latter can hardly be accepted as sufficient. The disease is probably due to germs. In the heat of the day these are carried high; at night they fall. They enter the body through vascular walls, and poison as really as arsenic. The limit of the power of the microscope, depending on the constitution of light, enables us to define objects $\frac{1}{100000}$ of an inch apart. But this is only half way to the ultimate molecules of matter, and a particle one-tenth of $\frac{1}{100000}$ of an inch in diameter may contain a million of molecules. Such a cause might poison and alter the blood most vitally without our being able to see the process. We want to administer something powerfully oxidizing in character, to wash or cleanse the body of the remains of this disease. We should administer first a diuretic. The acetate or the citrate of potassa is useful if quinine alone has failed. But, if they are not, a course of nitric acid may relieve for years.

6. *Pneumo-hydrothorax.*

June 6. A young man, rather small, aged twenty, a teamster, with bright dark eyes, began to cough a year ago. A month later he spat blood. He has expectorated, has had shortness of breath and night sweats ever since. Now respiration is rapid without dyspnœa. There is considerable abdominal movement, and but little thoracic. The right side is resonant down to the lower edge of the ribs. The liver is either contracted or displaced. There is too much air there; but this does not tell what has happened. By auscultation an amphoric sound is obtained. This is significant. But another rarer phenomenon is there. If we shake him a sound of splashing proceeds from the chest. This proves that both air and fluid exist together in the same compartment. Probably in the beginning he had pneumonia at the apex of the right lung, resulting in caseous deposits which broke down, cough and finally a rupture of the abscess into the pleural cavity.

*Examinations post-mortem.*—The case terminated suddenly. The patient seemed to be as well as usual during the week. He rose very early on June 13th and went to the bath room, then he returned quietly to bed. That is the last that was seen of him alive. When the nurse called him to get up he was dead. Both lungs were tuberculous. An old abscess of large size at the apex of the right lung, and a ragged opening at the top into the pleural cavity. The walls of the pleural sac were covered with discolored granulations. The cavity was partially filled with a dark, floculent and rather offensive liquid. The liver was crowded down. A mass of coagulated fibrine extended out of the heart into the trunks of the larger arteries. It is probable that death occurred as a consequence of arrest of the circulation. The fault was in the failure of the heart itself. There was at first some diminution of its force, the effect of innutrition of the nerve centers. More work had to be done, but the rate was not sufficient to keep the blood from coagulating. Fibrine began to separate, increasing the difficulty. The blood failed still more to reach the brain. Finally, as a result of this vicious circle, we may say the immediate cause of death was syncope.

7. *Bright's Disease and Degeneration of the Liver.*

Dr. L. introduced a sample of urine for analysis. It is not acid. It looks very turbid and high colored. In process of warming it begins to grow transparent, and when heated it becomes clear. Its turbidity is due to the presence of earthy phosphates, kept in solution by acid phosphates. When boiled it becomes turbid again. Nitric acid only increases the turbidity, showing that it is charged with albumen. A drop of dilute sulphuric acid poured on a thin stratum of this urine on a plate causes a violet color to play over the surface — a proof that bile pigment is present.

Now observe the patient. A finely built man, aged fifty-three; admitted yesterday; sick three months; skin pale and shining; legs, thighs, scrotum and hips all pitting on pressure. Resonance on the right side extends down to the nipple, but immediately below it is lost. The abdomen is flat, yet the peritoneal cavity contains fluid. The region of the stomach is firm, presenting a solid resisting mass, with shelf-like edges extending very near to the umbilicus. Percussion produces a flat sound all over the abdomen, except the space occupied by the colon; but deep resonance may be perceived behind the tumor. Only one thing can be the cause of this dullness — that is an enlarged liver. The urine pointed to disease of the kidney; besides, his peculiar whiteness would indicate the same thing. Simple inflammation of the kidney, some think, may be cured by $\frac{1}{32}$ of a grain of the bichloride of mercury and two grains of the iodide of potassium, three times daily, during several months. In general, put the patient to bed and keep him warm. If the house be heated by a furnace, it will be a good plan to give him a hot-air bath repeatedly, making him occupy a chair over a register enclosed in a little tent. The Turkish bath is useful. Give jalap and salts, or gamboge and colocynth. But remember that occasionally these remedies may take the patient out of the world by purging. Elaterium has its victims. In such ways, if there be no contra-indication, the skin and the bowels may be got to do the work of the kidney till the dropsy has subsided.

But how about the enlargement which we found? If this were an abscess it should give a sense of fluctuation. There is none. There should also be hectic fever and sweating. These do not exist. Cystic enlargements are generally projecting, distinctly globular and fluctuating. But there is nothing of the kind. In short, the history and the symptoms point pretty decidedly to malignant disease of the liver. It is however a good case to illustrate the uncertainties of diagnosis, for it is entirely possible that that may be amyloid degeneration of the liver and kidneys, or it may be fatty degeneration. We have simply a mass of probabilities from which to make a selection. We may therefore be prepared for differences of opinion. Different minds are affected differently by sets of symptoms. If you ask a positive opinion of what we have, then I must tell you I don't know. We shall wait to see. Examination after death, June 20, disclosed cancer of the liver.

